# Performance and diagnostic usefulness of commercially available enzyme linked immunosorbent assay and rapid kits for detection of HIV, HBV and HCV in India

**DOI:** 10.1186/1743-422X-9-290

**Published:** 2012-11-26

**Authors:** Susmita Maity, Srijita Nandi, Subrata Biswas, Salil Kumar Sadhukhan, Malay Kumar Saha

**Affiliations:** 1 NACO- National Reference Laboratory, National Institute of Cholera and Enteric Diseases, Kolkata, 700010, India

**Keywords:** Kit evaluation, HIV, HBV, HCV, Sera panel, ELISA, Rapid assay, Sensitivity, Specificity

## Abstract

**Background:**

HIV, HBV and HCV pose a major public health problem throughout the world. Detection of infection markers for these agents is a major challenge for testing laboratories in a resource poor setting. As blood transfusion is an important activity saving millions of live every year, it also carries a risk of transfusion transmissible infections caused by these fatal blood borne pathogens if the quality of testing is compromised. Conventional ELISA is regarded as the mostly used screening technique but due to limitations like high cost, unavailability in many blood banks and testing sites, involvement of costly instruments, time taking nature and requirement of highly skilled personnel for interpretation, rapid tests are gaining more importance and warrants comparison of performance.

**Results:**

A comparative study between these two techniques has been performed using commercially available diagnostic kits to assess their efficacy for detection of HIV, HBV and HCV infections. Rapid kits were more efficient in specificity with synthetic antigens along with high PPV than ELISA in most cases. Comparison between different ELISA kits revealed that Microlisa HIV and Hepalisa (J. Mitra & Co. Pvt. Ltd.); ERBA LISA HIV1 + 2, ERBA LISA Hepatitis B and ERBA LISA HCV (Transasia Bio-medicals Ltd.) gives uniform result with good performance in terms of sensitivity, specificity, PPV, NPV and efficiency, whereas, Microlisa HCV (J. Mitra & Co. Pvt. Ltd.), Microscreen HBsAg ELISA and INNOVA HCV (Span Diagnostics Ltd.) did not perform well. Rapid kits were also having high degree of sensitivity and specificity (100%) except in HIV Comb and HCV Comb (J. Mitra & Co. Pvt. Ltd.). The kit efficiency didn’t vary significantly among different companies and lots in all the cases except for HCV ELISA showing statistically significant variation (p < 0.01) among three kit types.

**Conclusions:**

ELISA is a good screening assay for markers of HIV, HBV and HCV infections. Rapid tests are useful for further detection of false positive samples. ELISA seems the appropriate assay in blood bank. For availability of quality commercial diagnostic assays, evaluation of kit may be helpful.

## Background

Human immunodeficiency virus (HIV), hepatitis B virus (HBV) and hepatitis C virus (HCV) are the most threatening blood borne pathogens which have proved to be major risk factors for transfusion transmissible infections in human population. Blood transfusion is an important activity that saves millions of live every year [1]. But still it carries risk of transfusion associated diseases like hepatitis, acquired immunodeficiency syndrome (AIDS) and some other blood borne sexually transmitted diseases [2,3]. About 350 millions of patients are suffering from hepatitis B all over the world [3,4]. In India, 2–3 million individuals have been reported to be infected with HIV and transmission of this infection has been shown to be strongly associated with other sexually transmitted infections (STIs) and sexual behavior [5]. Hepatitis B and C causes severe complications after transfusion of contaminated blood [6-12]. It has been estimated that the global prevalence of Hepatitis C virus (HCV) infection is around 2%, with 170 million persons chronically infected with the virus and 3 to 4 million persons newly infected each year [13]. Transmission of hepatitis C through intravenous and percutaneous drug usage is a significant problem in northeast India and the prevalence of HCV was an alarming 92% among 77 IV drug abusers from Manipur [14]. In two studies from Mumbai the prevalence of HCV in multiple transfused thallasemics was 16.7% and 17.5% respectively [15,16]. In multi-transfused children with varied diagnosis, the prevalence of HCV was 13% reported from Kolkata [17]. Occurrence of HIV has also been increased significantly in the developing countries [18,19]. So there is an acute need for testing of markers for HIV1 and 2, Hepatitis B, Hepatitis C and other sexually transmitted diseases (STD). Conventional enzyme linked immunosorbent assay (ELISA) is most referred screening technique [3] and possess an accuracy of about 99.9% [20,21] with improved sensitivity but some of the kits reported to have lower specificity [22]. Additionally, the method is laborious, time taking and needs proficient skill to perform and also not available in many blood bank/testing sites. Comparatively, rapid tests are easier, quicker and require less skill to perform. There is no requirement of any instruments also. Present study deals with the evaluation of performance as well as usefulness of both ELISA and rapid assay for detection of three major blood borne pathogens namely HIV, HBV and HCV using separate panel-sera for each. Three most commonly available ELISA and rapid kits (three lots for each kit- type) were evaluated of which two ELISA and rapid kits each were of same manufacturer.

## Results

In current study, 300 samples (100 samples/lot, 3 lots evaluated for each kit) were tested for each set of evaluation of both ELISA and rapid kits for HIV, hepatitis B surface antigen (HBsAg) and HCV and the results were summarized in Table 1. It is evident that Microlisa HIV, ERBA LISA HIV 1 + 2, ERBA LISA Hepatitis B, ERBA LISA HCV and Hepalisa - HBsAg gives uniform results by identifying all panel samples correctly with good performance (100% sensitivity, specificity, PPV, NPV and efficiency) as compared to other ELISA kits (Table 2). Enzaids HIV 1 + 2 also had similar performance except in one lot where specificity was 98.9%. Performance of Microlisa HCV, Microscreen HBsAg ELISA and INNOVA HCV are not satisfactory with higher number of false positive results (Table 1) and subsequent reduced specificity, but all of the ELISA kits are capable of detecting positive samples correctly that also reflected in kit sensitivity (100%). It is also evident from the present report that rapid kits have good specificity and sensitivity (100%) except HIV Comb (mean sensitivity 98.3%) and HCV Comb (mean sensitivity 95.5%) showing poor performance (Table 3) with some false negative results for low positive sera. In each panel of HIV, HBV and HCV four low positive sera were included. HIV comb was unable to detect 2 low positives in one lot, whereas, HCV comb gives false negatives in two lots (2 false negatives in lot 1 and 4 false negatives in lot 2; all were low positive sera).

**Table 1:** Performance of HIV, HBsAg and HCV kits with known Panel Sera

**Test parameter**	**Status of panel sera**			**Kit performance**												
	**Total Sera**	**Positive Sera**	**Negative Sera**	**Lot**	**ELISA**						**Rapid Test**					
					**J Mitra & Co. Pvt Ltd**		**SPAN Diagnostics Ltd.**		**Transasia Bio medicals Ltd.**		**J Mitra & Co. Pvt Ltd**		**SPAN Diagnostics Ltd.**		**Standard Diagnostics, INC**	
					**Positive**	**Negative**	**Positive**	**Negative**	**Positive**	**Negative**	**Positive**	**Negative**	**positive**	**Negative**	**Positive**	**Negative**
HIV	100	40	60	LOT:1	40	60	42	58	40	60	40	60	40	60	40	60
				LOT:2	40	60	40	60	40	60	40	60	40	60	40	60
				LOT:3	40	60	40	60	40	60	38	62	40	60	40	60
HBsAg	100	40	60	LOT:1	40	60	42	58	40	60	40	60	40	60	40	60
				LOT:2	40	60	40	60	40	60	40	60	40	60	40	60
				LOT:3	40	60	42	58	40	60	40	60	40	60	40	60
HCV	100	44	56	LOT:1	51	49	50	50	44	56	42	58	44	56	44	56
				LOT:2	51	49	46	54	44	56	40	60	44	56	44	56
				LOT:3	50	50	46	54	44	56	44	56	44	56	44	56

Three different set of 100 member sera panels were used for evaluation of commercially available ELISA and rapid kits in India, for HIV, HBsAg and HCV.

**Table 2:** Performance characteristic of HIV, HBsAg and HCV ELISA kits used for comparative evaluation

**Test Parameter**	**Kit Company**	**Lot**	**Kit Performance**									
			**Sensitivity**		**Specificity**		**PPV**		**NPV**		**Efficiency**	
			**(%)**	**%Mean (95%CI)**	**(%)**	**%Mean (95%CI)**	**(%)**	**%Mean (95%CI)**	**(%)**	**%Mean (95%CI)**	**(%)**	**%Mean (95%CI)**
**HIV**	J. Mitra & Co. Pvt. Ltd.	1	100	100 (100–100)	100	100 (100–100)	100	100 (100–100)	100	100 (100–100)	100	100 (100–100)
		2	100		100		100		100		100	
		3	100		100		100		100		100	
	SPAN Diag. Ltd.	1	100	100 (100–100)	96.7	98.9 (96.8-100.9)	95.2	98.4 (95.8-101.0)	100	100 (100–100)	98.0	99.3 (98.3-100.4)
		2	100		100		100		100		100	
		3	100		100		100		100		100	
	Transasia Bio medicals Ltd.	1	100	100 (100–100)	100	100 (100–100)	100	100 (100–100)	100	100 (100–100)	100	100 (100–100)
		2	100		100		100		100		100	
		3	100		100		100		100		100	
**HBsAg**	J. Mitra & Co.Pvt. Ltd.	1	100	100 (100–100)	100	100 (100–100)	100	100 (100–100)	100	100 (100–100)	100	100 (100–100)
		2	100		100		100		100		100	
		3	100		100		100		100		100	
	SPAN Diag. Ltd.	1	100	100 (100–100)	96.7	97.8 (96.0 −99.6)	95.2	96.8 (94.2 −99.4)	100	100 (100–100)	98.0	98.7 (97.6-99.7)
		2	100		100		100		100		100	
		3	100		96.7		95.2		100		98.0	
	Transasia Bio medicals Ltd.	1	100	100 (100–100)	100	100 (100 −100)	100	100 (100–100)	100	100 (100–100)	100	100 (100–100)
		2	100		100		100		100		100	
		3	100		100		100		100		100	
**HCV**	J. Mitra & Co.Pvt. Ltd.	1	100	100 (100–100)	87.5	88.1 (87.1 −89.1)	86.3	86.9 (86.0 −87.8)	100	100 (100–100)	93.0	93.3 (92.8-93.9)
		2	100		87.5		86.3		100		93.0	
		3	100		89.3		88.0		100		94.0	
	SPAN Diag. Ltd.	1	100	100 (100–100)	89.3	94.0 (90.2 −97.8)	88.0	93.1 (89.0 −97.2)	100	100 (100–100)	94.0	96.7 (94.5-98.8)
		2	100		96.4		95.7		100		98.0	
		3	100		96.4		95.7		100		98.0	
	Transasia Bio medicals Ltd.	1	100	100 (100–100)	100	100 (100–100)	100	100 (100–100)	100	100 (100–100)	100	100 (100–100)
		2	100		100		100		100		100	
		3	100		100		100		100		100	

CI- Confidence Interval, PPV-Positive Predictive Value, NPV-Negative Predictive Value.

**Table 3:** Performance characteristic of HIV, HBsAg and HCV rapid kits used for comparative evaluation

**Test Parameter**	**Kit Company**	**Lot**	**Kit Performance**									
			**Sensitivity**		**Specificity**		**PPV**		**NPV**		**Efficiency**	
			**(%)**	**%Mean (95%CI)**	**(%)**	**%Mean(95%CI)**	**(%)**	**%Mean (95%CI)**	**(%)**	**%Mean (95%CI)**	**(%)**	**%Mean (95%CI)**
**HIV**	J. Mitra & Co. Pvt. Ltd.	1	100	98.3 (95.7-101.0)	100	100 (100–100)	100	100 (100–100)	100	98.9 (97.2-100.6)	100	99.3 (98.3-100.4)
		2	100		100		100		100		100	
		3	95.0		100		100		96.8		98.0	
	SPAN Diag. Ltd.	1	100	100 (100–100)	100	100 (100–100)	100	100 (100–100)	100	100 (100–100)	100	100 (100–100)
		2	100		100		100		100		100	
		3	100		100		100		100		100	
	Standard. Diag. Inc.	1	100	100 (100–100)	100	100 (100–100)	100	100 (100–100)	100	100 (100–100)	100	100 (100–100)
		2	100		100		100		100		100	
		3	100		100		100		100		100	
**HBsAg**	J. Mitra & Co. Pvt. Ltd.	1	100	100 (100–100)	100	100 (100–100)	100	100 (100–100)	100	100 (100–100)	100	100 (100–100)
		2	100		100		100		100		100	
		3	100		100		100		100		100	
	SPAN Diag. Ltd.	1	100	100 (100–100)	100	100 (100–100)	100	100 (100–100)	100	100 (100–100)	100	100 (100–100)
		2	100		100		100		100		100	
		3	100		100		100		100		100	
	Standard. Diag. Inc.	1	100	100 (100–100)	100	100 (100–100)	100	100 (100–100)	100	100 (100–100)	100	100 (100–100)
		2	100		100		100		100		100	
		3	100		100		100		100		100	
**HCV**	J. Mitra & Co. Pvt. Ltd.	1	95.5	95.5 (91.2-99.7)	100	100 (100–100)	100	100 (100–100)	96.6	96.6 (93.5-99.8)	98.0	98.0 (96.1-99.8)
		2	90.9		100		100		93.3		96.0	
		3	100		100		100		100		100	
	SPAN Diag. Ltd.	1	100	100 (100–100)	100	100 (100–100)	100	100 (100–100)	100	100 (100–100)	100	100 (100–100)
		2	100		100		100		100		100	
		3	100		100		100		100		100	
	Standard. Diag. Inc.	1	100	100 (100–100)	100	100 (100–100)	100	100 (100–100)	100	100 (100–100)	100	100 (100–100)
		2	100		100		100		100		100	
		3	100		100		100		100		100	

CI- Confidence Interval, PPV-Positive Predictive Value, NPV-Negative Predictive Value.

Present study revealed a better performance in rapid kits of Standard Diagnostics Inc. and Span Diagnostics Ltd. in terms of specificity and sensitivity than J. Mitra & Co. Pvt. Ltd. except for HBsAg rapid kits where performance of all kits is equally good. Mean specificity of HIV and HBsAg ELISA kits manufactured by SPAN Diagnostics Ltd. are relatively lower (<100%) than Transasia Biomedicals Ltd. and J. Mitra and Co. Pvt. Ltd. (100%) except for HCV ELISA kits where lower specificity with many false positives found in Microlisa HCV. A total of 20 false positives with 88.1% mean specificity observed in Microlisa HCV which is quite poor performance in terms of detection of infection. Innova HCV and Microscreen HBsAg ELISA also had shown false positive results. A 2-way ANOVA test also used to assess the statistical variability in efficiency values due to ‘companies’ and ‘lots’ and this statistical analysis decipher that the efficiency of three commercially available rapid kits of all three categories (HIV, HBsAg and HCV) didn’t vary significantly (p > 0.05) among the companies as well as in the lots. Same observation also recorded in HIV and HBsAg ELISA kits where no significant variation found in efficiency of three kit types and among the lots (p > 0.05). Whereas, in HCV ELISA, kit efficiency varies significantly among three different kit types (p < 0.01, F value =20.0, df: η_1_ = 2, η_2_ = 4) but no significant variation found in lots.

## Discussion

Detection of HIV, HBV and HCV infections and diagnosis is mainly based on immunological assays among which ELISA and rapid tests are most common and widespread methods [23,24]. An important problem encountered at this point is the discordance between the results of two assays [22], which can be resolved depending on the availability of suitable kits. Hence, kit evaluation gains importance for determining the diagnostic kits of better performance. Though ELISA assay shows a high degree of sensitivity, it is costly and time taking job, so rapid tests become a good alternative for ELISA in blood banks and other testing laboratories in course of time [25]. Moreover, uses of synthetic antigens in some rapid kits have increased the specificity [25]. Performance of rapid test would be satisfactory with a high PPV and lower degree of false negatives [23].

This study revealed a higher PPV in rapid tests along with better efficiency (100%) than ELISA in most of the cases except for HIV comb and HCV Comb- J. Mitra & Co. Pvt. Ltd. revealed 99.3% and 98.0% efficiency respectively. Although for a diagnostic test, the PPV and NPV will change as the prevalence of the target condition changes in the tested population. Thus, the J. Mitra & Co. HCV ELISA kit would project a PPV of 6.6%, 41.7% and 22.2% for global population with 0.8% HIV, 5% HBV and 2% HCV prevalence respectively. 4th generation HCV-comb with unique combination of modified HCV antigens have failed to show better result in this study with lowest efficiency and sensitivity (95.5%) than two other HCV rapid kits, therefore use of this type of kit in blood banks should be avoided.

HIV comb with recombinant antigens have also shown much less sensitivity (98.3%) than other HIV rapid kits evaluated, whereas, the uses of synthetic peptides along with recombinant antigen have given an added advantage to Combaids RS Advantage (22) with a better performance than HIV-comb. SD BIOLINE HIV 1/2 3.0 Rapid have also shown better result with 100% sensitivity. So, considering these parameters, HIV comb is not an appropriate kit for blood banks and ICTCs in terms of its lower sensitivity than other HIV rapid kits.

The HIV ELISA kits manufactured by SPAN Diagnostics Ltd. were revealed poor performance than the other HIV ELISA kits in terms of specificity and overall efficiency of those kits was also poor (99.3%). This was also true for Microscreen HBsAg ELISA kits (98.7%) but, in case of HCV ELISA kits, HCV Microlisa were also shown lower efficiency (93.3%) along with INNOVA HCV ELISA (96.7%).

Present study also showed lot to lot variation in performance of some kits. Mostly it was found in HCV Microlisa, Enzaids HIV 1 + 2 ELISA, Micro screen HBsAg ELISA, Innova HCV ELISA, HIV-EIA Comb and HCV-Comb.

It was found that the kit efficiency didn’t varies significantly among three different kit companies and their lots in all the cases except for HCV ELISA showing statistically significant variation (p < 0.01) among three kit types. According to present study, Hepacard, Combaids RS- Advantage-HIV, Crystal HBsAg, Signal HCV, SD BIOLINE HIV Â½ 3.0 Rapid, SD BIOLINE HBsAg Rapid and SD BIOLINE HCV Rapid are the recommended rapid kits demonstrating good performance whereas, Microlisa HIV, ERBA LISA HIV1 + 2, ERBA LISA Hepatitis B, ERBA LISA HCV and Hepalisa (HBsAg) are most acceptable ELISA kits with no false positive results. It is needed to mention that present study was performed with serum samples; therefore, performance of rapid tests may vary if whole blood sample is used as done in the point of care. As per overall results decipher, rapid tests are more acceptable than ELISA for its specificity But, ELISA proved to be more superior to rapid tests in terms of sensitivity. In case of diagnosis of infectious disease, discordant results may have serious consequences among the patients as it causes unnecessary mental stress and tension. For proper diagnosis of infection as well as disease management and prevention, identification of appropriate test kit is necessary. According to this kit evaluation, a higher number of false positive results obtained in Microlisa HCV, Innova HCV ELISA and Microscreen HBsAg ELISA which is really a matter of concern. Again, false negative results leave a threat of silent transmission and spreading of diseases among people as produced by rapid HCV-comb and HIV-comb assay and also create an urge for sensitive assays like ELISA. Therefore, in resource poor setting where ELISA is unavailable, practice of using rapid kits for blood banks may lead to spread of the deadly infection.

The HIV and HCV kits detect only IgG and thus miss the IgM which is a critical marker of early infection. Testing with these kits might lead to false negative for samples of recent infection. Therefore, the tests are subject to the serious limitation which might affect a small fraction of samples analyzed. Kits able to capture both IgM and IgG needed to be developed to reduce the chance of false negative and eventually helping in fight against the pathogens.

## Conclusions

Commercially available ELISA is good for screening of HIV, HBV and HCV infections but rapid tests, as it is having higher specificity, may be used further for detection of false positive samples. In blood bank perspective, ELISA seems the appropriate assay for the screening. Improvement of sensitivity of rapid kit, which is attainable as evident in most of the kits evaluated, will help resource poor setting. A regular mechanism of kit evaluation will help ensuring availability of quality commercial kits.

## Methods

The study was carried out at NACO-National Reference Laboratory at National Institute of Cholera and Enteric Diseases, Indian Council of Medical Research, Kolkata, India, which is a designated laboratory for evaluation of diagnostic kits. Three different set of 100 member sera panels were used for evaluation of commercially available ELISA and rapid kits in India, for HIV, HBsAg and HCV. Characterization of sera panel was done by evaluation of individual serum by two commercially available ELISA kits, a rapid assay and a confirmatory western blot assay / recombinant immuno-blot assay (RIBA)/ PCR as detailed in Table 4. Sample reactive in all the assays were defined as positive member in the panel and a negative member was non-reactive in all the assays. All three sera panels contain low positive sera that have shown uniform results in all assays used for their validation.

**Table 4:** Characterization of panel sera for HIV, HBV and HCV

**Name of Panel**	**Test Details**		
	**ELISA**	**Rapid Test**	**Confirmatory Test**
HIV Sera Panel	ELISA 1: Vironostika HIV Ag/ Ab (BIOMERIEUX) ELISA 2: Genescreen HIV 1/ 2, version 2. (Bio-Rad Laboratories)	HIV Tri-Dot (J. Mitra & Co. Pvt. Ltd.)	Western Blot: NEW LAV BLOT 1 (Bio-Rad Laboratories)
HBV Sera Panel	ELISA 1: Hepanostika HBsAg Ultra (BIOMERIEUX) ELISA 2: MONOLISA HBsAg ULTRA (Bio-Rad Laboratories)	Immunocomb II, HBsAg (ORGENICS)	MONOLISA HBsAg CONFIRMATION (Bio-Rad Laboratories)
HCV Sera Panel	ELISA 1: Ortho 3.0 Enhanced SAVe (Ortho Clinical Diagnostics) ELISA 2: Monolisa anti-HCV Plus (Bio-Rad Laboratories)	HCV TRI-DOT (J. Mitra & Co. Pvt. Ltd.)	Western Blot: RIBA (Recombinant Immuno-Blot assay)- CHIRON RIBA HCV 3.0 SIA

Commercially available ELISA kits evaluated were Microlisa- HIV, Hepalisa for HBsAg and HCV Microlisa- manufactured by J. Mitra & Co. Pvt. Ltd, New Delhi, India; Enzaids HIV 1 + 2 ELISA, Micro screen HBsAg ELISA and Innova HCV ELISA - manufacturer Span Diagnostics Ltd., Surat, India and ERBA LISA HIV 1 + 2, ERBA LISA HEPATITIS B and ERBA LISA HEPATITIS C- manufacturer Transasia Biomedicals Ltd. Mumbai, India. The Rapid kits evaluated were HIV-EIA Comb, Hepacard and HCV-Comb- J. MITRA & CO. PVT. LTD, New Delhi, India; Combaids RS- Advantage-HIV, Crystal HBsAg and Signal HCV Ver. 2.0- Span Diagnostics Ltd. Surat, India and SD BIOLINE HIV Â½ 3.0 Rapid, SD BIOLINE HBsAg Rapid and SD BIOLINE HCV Rapid- manufactured by Standard diagnostics INC, Haryana, India. The kits selected in this study were most frequently used in ICTCs and blood banks for detection of HIV, HBsAg and HCV as well as were approved by Central Drug Standard Control Organization for Blood Banks [26]. Three lots for each ELISA and rapid kit-types have been evaluated and their specifications were detailed in Table 5. Manufacturer’s instructions and kit literature were strictly followed for validation of assay. Performance of kits for each lot was evaluated in terms of sensitivity, specificity, positive predictive value, negative predictive value and efficiency which can be defined as follows:

● Sensitivity = It is the ability of an assay to detect truly infected individuals and very small amounts of analyte. It can be calculated by following formula:

$$\text{Sensitivity}=\left[\text{True Positives}/\left(\text{True Positives}+\text{False Negatives}\right)\right]\;\text{X}\;100$$

● Specificity = It is the ability of an assay to correctly identify all the uninfected individuals and there should be no false positives. It can be calculated by following formula:

$$\text{Specificity}=\left[\text{True Negatives}/\left(\text{True Negatives}+\text{False Positives}\right)\right]\;\text{X}\;100$$

● Positive Predictive Value (PPV) = It is the ability of a test to identify actually infected individuals among all persons giving a positive result with the kit being used. It is calculated by following formula:

$$\text{PPV}=\left[\text{True Positives}/\left(\text{True Positives}+\text{False Positives}\right)\right]\;\text{X}\;100$$

● Negative Predictive Value (NPV) = It is the ability of a test to identify correctly the real non infected individuals among all persons giving a negative result with the kit being used. It is calculated by following formula:

$$\text{NPV}=\left[\text{True Negatives}/\left(\text{True Negatives}+\text{False Negatives}\right)\right]\;\text{X}\;100$$

● Efficiency = It is the overall ability of a test to correctly identify all positives as positive and all negatives as negative. This is also referred to as ‘accuracy’. It is calculated as follows

$$\text{Efficiency}=\left[\left(\text{True positives}+\text{True Negatives}\right)/\left(\text{True Positives}+\text{False Negatives}+\text{True Negatives}+\text{False Positives}\right)\right]\;\text{X}\;100$$

**Table 5:** Specification characteristics of ELISA and rapid kits used for evaluation

**Kit Type**	**Kit Name**	**Company Name**	**Kit Specifications**	**Detectable Ag/Ab**
HIV ELISA	Microlisa-HIV	J. Mitra & Co. Pvt Ltd.	Indirect ELISA, recombinant antigens for HIV-1: gp41, C-terminus of gp120; for HIV-2: gp36 used in MWP.	IgG
	Enzaids HIV 1 + 2 ELISA	SPAN Diagnostics Ltd.	Indirect ELISA, a combination of recombinant and synthetic peptides, derived from immuno-dominant regions of HIV-1 (gp120, gp41) and HIV-2 (gp36) used in MWP.	IgG
	ERBA LISA HIV 1 + 2	Transasia Biomedicals Ltd.	Indirect ELISA, synthetic peptide of HIV 1 & 2 coated onto MWP.	IgG
HBsAg ELISA	Hepalisa	J. Mitra & Co. Pvt Ltd.	Direct sandwich, microwell plates coated with immobilized HBsAg antibodies	HBsAg
	Microscreen HBsAg ELISA	SPAN Diagnostics Ltd.	Direct non-competitive ELISA, microwell plates coated with immobilized HBsAg antibodies	HBsAg
	ERBA LISA HEPATITIS B	Transasia Biomedicals Ltd.	Sandwich method, microwell plates coated with immobilized HBsAg antibodies	HBsAg
HCV ELISA	Microlisa HCV	J. Mitra & Co. Pvt Ltd.	Indirect ELISA, 3rd generation tests and utilizes recombinant antigens from CORE, E1, E2, NS3, NS4 and NS5 genomic regions	IgG
	Innova HCV ELISA	SPAN Diagnostics Ltd.	Indirect ELISA, 3rd generation test with highest sensitivity (100%) ensured by use of recombinant antigens from core, E1, E2, NS3, NS4 & NS5 genomic regions.	IgG
	ERBA LISA HEPATITIS C	Transasia Biomedicals Ltd.	Indirect ELISA, 3rd generation tests, utilizes a combination of antigens from CORE, NS3, NS4 and NS5 genomic regions	IgG
HIV RAPID	HIV Comb	J. Mitra & Co. Pvt Ltd.	Dot Immunoassay, utilizes recombinant antigens for HIV-1: gp41, C-terminus of gp120; for HIV-2: gp36.	IgG
	Combaids-RS Advantage	SPAN Diagnostics Ltd.	Dot Immunoassay, a combination of recombinant and synthetic peptides from immuno-dominant regions of HIV-1 (gp120, gp41) & HIV-2 (gp36).	IgG
	SD BIOLINE HIV Â½ 3.0	Standard Diagnostics, INC	Rapid immunochromatographic tests, membrane strip precoated with recombinant HIV-1 capture antigen (gp41, p24) on test band 1 region and with recombinant HIV-2 capture antigen (gp36) on test band 2.	IgG
HBsAg RAPID	HEPACARD	J. Mitra & Co. Pvt Ltd.	One step, rapid, visual and qualitative immunochromatographic assay based on the antigen capture or “sandwich” principle, utilizes monoclonal antibodies for HBsAg.	HBsAg
	Crystal HBsAg	SPAN Diagnostics Ltd.	One step, rapid visual immunochromatographic tests for qualitative detection of Hepatitis B surface antigen (HBsAg), utilizes highly specific monoclonal antibodies ensure no cross reactivity with HAV and HCV	HBsAg
	SD BIOLINE HBsAg	Standard Diagnostics, INC	One step, rapid, visual and qualitative immunochromatographic assay, membrane strip precoated with mouse monoclonal anti-HBs capture antibody on test band.	HBsAg
HCV RAPID	HCV-Comb	J. Mitra & Co. Pvt Ltd.	4th generation Dot Immunoassay, utilizes a unique combination of modified HCV antigens from the putative core, NS3, NS4 & NS5 genomic regions	IgG
	Signal HCV	SPAN Diagnostics Ltd.	3rd generation immuno-dot test comprising of recombinant antigens from core, E1,E2,NS3,NS4 & NS5 genomic regions of HCV	IgG
	SD BIOLINE HCV	Standard Diagnostics, INC	One step, rapid, visual and qualitative immunochromatographic assay, membrane strip precoated with recombinant HCV capture antigen (CORE, NS3, NS4 and NS5) on test band.	IgG

The evaluation process maintained unbiased following a double-blind procedure (separate personnel adopted for pre-analytical and analytical/testing sections and finally both of them involved in result verification for test validation, but final analysis of result was done independently by the Lab In-charge). Information regarding status of panel samples has been restricted from testing personnel.

Sera used in this study were obtained anonymously from volunteer attendees of the Integrated Counseling and Testing Centre following informed consent from each individual as per protocol approved by the Institutional Ethics Committee, National Institute of Cholera and Enteric Diseases.

### Statistical analysis

Confidence Interval (CI) was used to address precision of the proportion estimates and the degree of confidence was set to 95% [22]. Analysis of variance (ANOVA) also has been performed among different kit companies as well as in lots to enquire the significance of variations in kit efficiency.

## Abbreviations

HIV: Human Immunodeficiency Virus; HBV: Hepatitis B Virus; HCV: Hepatitis C Virus; AIDS: Acquired Immunodeficiency Syndrome; STI: Sexually Transmitted Infections; STD: Sexually Transmitted Disease; ELISA: Enzyme Linked Immuno Sorbent Assay; HBsAg: Hepatitis B Surface Antigen; OD: Optical Density; NACO: National AIDS Control Organization.

## Competing interest

The authors declare that they have no competing interests.

## Authors’ contributions

SM, SN and MKS conceived the study. All authors performed combined analysis, contributed in drafting, critically reviewed and approved the final manuscript.
